# Molecular Mechanism of Oocyte Activation in Mammals: Past, Present, and Future Directions

**DOI:** 10.3390/biom14030359

**Published:** 2024-03-17

**Authors:** Hibiki Sugita, Shunsuke Takarabe, Atsuko Kageyama, Yui Kawata, Junya Ito

**Affiliations:** 1 Laboratory of Animal Reproduction, Graduate School of Veterinary Sciences, Azabu University, Sagamihara 252-5201, Japan; 2 School of Veterinary Medicine, Azabu University, Sagamihara 252-5201, Japan

**Keywords:** phospholipase, mammals, fertilization, oocyte activation, sperm factor, Zn^2+^, Ca^2+^

## Abstract

During mammalian fertilization, repetitive intracellular Ca^2+^ increases known as Ca^2+^ oscillations occur. These oscillations are considered crucial for successful fertilization and subsequent embryonic development. Numerous researchers have endeavored to elucidate the factors responsible for inducing Ca^2+^ oscillations across various mammalian species. Notably, sperm-specific phospholipase C zeta (PLCζ) emerged as a prominent candidate capable of initiating Ca^2+^ oscillations, particularly in mammals. Genetic mutation of PLCζ in humans results in the absence of Ca^2+^ oscillations in mouse oocytes. Recent studies further underscored PLCζ’s significance, revealing that sperm from PLCζ-deficient (*Plcz1^−/−^*) mice fail to induce Ca^2+^ oscillations upon intracytoplasmic sperm injection (ICSI). Despite these findings, observations from in vitro fertilization (IVF) experiments using *Plcz1^−/−^* sperm revealed some residual intracellular Ca^2+^ increases and successful oocyte activation, hinting at potential alternative mechanisms. In this review, we introduced the current hypothesis surrounding oocyte activation in mammals, informed by contemporary literature, and probed into the enigmatic mechanisms underlying mammalian fertilization-induced oocyte activation.

## 1. The Discovery of PLCζ as a Factor Inducing Oocyte Activation

### 1.1. Essential Events of Mammalian Fertilization

Mammalian fertilization heralds a pivotal moment in the inception of life, which requires the ovulated oocyte and sperm [1]. Throughout this process, the sperm embark on a journey through the vaginal canal and uterus, ultimately reaching the oviductal ampullae, which serves as the primary site of fertilization in most mammalian species [2,3]. Meanwhile, oocytes expelled from the ovary remain arrested at metaphase during the second meiosis (MII), awaiting fertilization [4,5]. Preceding fertilization, sperm undergo a series of physiological and morphological transformations, including capacitation, the acrosome reaction (AR), and hyperactivation [6].

After the physiological and morphological transformations, the sperm trigger “oocyte activation”, which includes the exocytosis of cortical granules (CGs), resumption from the MII arrest, extrusion of the second polar body, and pronuclear formation (PN) [7,8,9]. Failure to induce oocyte activation at the time of fertilization results in the absence of the aforementioned biochemical and morphological changes, thereby inhibiting subsequent embryonic development [10]. Successful fertilization necessitates the fusion of a single sperm with a single oocyte [11]. The entry of two or more sperm into the oocyte leads to polyspermy [11]. Generally, both the zona reaction and a membrane block function prevent polyspermy [3].

**Figure 1:**
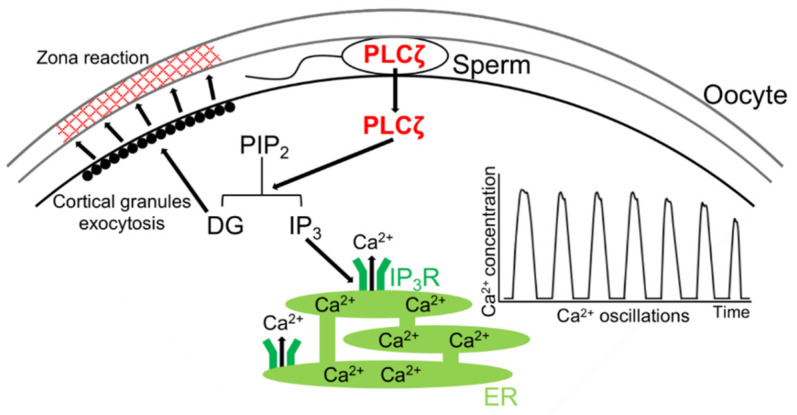
Mechanism of rise(s) in intracellular Ca^2+^ level via PLCζ during mammalian fertilization. After the sperm–oocyte fusion, PLCζ is released from the sperm into the oocyte. PLCζ hydrolyzes phosphatidylinositol 4,5-bisphosphate (PIP_2_) to inositol trisphosphate (IP_3_) and diacylglycerol (DG). DG induces the exocytosis of cortical granules, results in the zona reaction, and IP_3_ binds to the IP_3_ receptor (IP_3_R), which leads to the release of Ca^2+^ from the endoplasmic reticulum (ER).

Upon penetration of the oocyte, sperm induce the exocytosis of CGs [12]. The released CGs modify the protein structure within the zona pellucida, leading to the zona reaction [12] (Figure 1). Furthermore, physiological alterations to the oocyte’s membrane following sperm penetration serve as a barrier to prevent multiple sperm from entering the oocyte cytoplasm [13]. Polyspermy during fertilization results in the formation of multiple pronuclei and the generation of polyploid embryos, which typically fail to develop or perish shortly after implantation [14]. The arrest at the metaphase II (MII) stage is regulated by the cytostatic factor, which maintains the high activity of the maturation-promoting factor (MPF) consisting of cyclin-dependent protein kinase 1 and cyclin B [10]. Upon sperm entry into the oocyte during fertilization, cyclin B undergoes degradation, leading to the inactivation of MPF [10]. Consequently, chromosome decondensation ensues, facilitating pronuclear formation [15]. The inactivation of MPF requires calcium ions (Ca^2+^) for oocyte activation, as a rise in Ca^2+^ levels leads to MPF inactivation through the activation of Ca^2+^/calmodulin-dependent protein kinase gamma (CamKIIγ) and the degradation of early mitotic inhibitor 2 (Emi2) [16]. Therefore, an increase in Ca^2+^ ions within the oocyte is considered indispensable for oocyte activation during fertilization [16].

### 1.2. Ca^2+^ Oscillations

**Figure 2:**
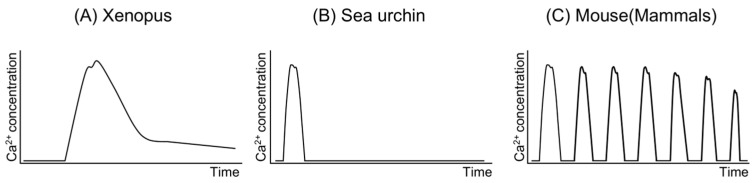
Representative Ca^2+^ release at fertilization in oocytes of several species.

Rises in Ca^2+^ concentration within the oocyte are observed during fertilization across all species investigated to date [17]. These Ca^2+^ increases occur as a single wave in some species, such as Xenopus and sea urchins [18,19,20,21], while in others, they manifest as repetitive waves [22,23,24] (Figure 2). In mammals like humans and mice, oocytes exhibit repetitive Ca^2+^ increases, known as “Ca^2+^ oscillations”, immediately following sperm–oocyte fusion [22,25,26,27], with variations in duration, amplitude, and frequency observed among species [26]. In mouse oocytes, the typical Ca^2+^ concentration is approximately 100 nM, peaking at just under 1000 nM, with each increase lasting approximately 0.5 min [25,28]. Ca^2+^ oscillations persist for about 4–5 h, ceasing around pronuclear formation [29,30,31]. Consequently, Ca^2+^ oscillations are regarded as a pivotal event for oocyte activation, particularly in mammals. Numerous researchers have endeavored to elucidate the mechanisms underlying Ca^2+^ oscillations [32,33]. Studies have demonstrated that a reduction in the number of Ca^2+^ oscillations during fertilization in mice results in aberrant gene expression in blastocysts and abnormal postnatal growth of offspring following embryo transfer of the blastocyst [34]. Nevertheless, whether Ca^2+^ oscillations are indispensable for mammalian fertilization remains unclear.

The significance of Ca^2+^ during mammalian fertilization has been underscored through various experiments. For instance, treatment with the Ca^2+^ chelator 1,2-bis-(O-aminophenoxy)-ethane-*N*,*N*,*N*’,*N*’,-tetraacetic acid (BAPTA) prevented the elevation of Ca^2+^ levels in mouse oocytes, resulting in inhibition of CG exocytosis, meiotic resumption, and pronuclear (PN) formation [25]. Conversely, treatments of mouse oocytes with Ca^2+^ ionophores, ethanol, or electrical stimulation led to an increase in Ca^2+^ concentration within the oocytes, inducing oocyte activation [35,36,37]. Following these treatments, oocytes were capable of forming PN and subsequently developing into blastocysts. These experiments unequivocally demonstrated that the elevation of Ca^2+^ levels in the oocyte is adequate to trigger oocyte activation in mammals. However, the activation rate of oocytes was not notably high [23,24], possibly due to the transient nature of the Ca^2+^ increase rather than the repetitive Ca^2+^ oscillations characteristic of mammalian fertilization [38]. Thus, these experiments established that in mice, repetitive increases in Ca^2+^ are indispensable for oocyte activation and subsequent embryonic development.

### 1.3. The Sperm Factor Theory and the Discovery of PLCζ

**Figure 3:**
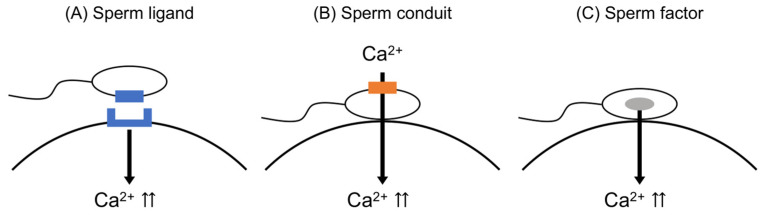
Hypotheses regarding the sperm-inducing Ca^2+^ release during mammalian fertilization.

Three hypotheses have been proposed regarding the mechanism of Ca^2+^ oscillations in the oocyte during fertilization (Figure 3). The first hypothesis, known as the “Sperm ligand theory”, suggests that receptors on the oocyte membrane bind to ligands on the sperm, leading to an increase in Ca^2+^. This process is believed to involve G proteins that activate phospholipase C isoforms (PLCs) within the oocyte [39]. The second hypothesis, termed the “Sperm conduit theory”, posits that extracellular Ca^2+^ enters the oocyte through the sperm when the sperm membrane fuses with the oocyte membrane [19,40]. However, the success of intracytoplasmic sperm injection (ICSI) has challenged these two hypotheses, as ICSI does not involve the fusion of sperm and oocyte membranes during fertilization. 

The third hypothesis, known as the “sperm factor (SF) theory”, is widely considered a plausible explanation for the induction of Ca^2+^ oscillations. It has been demonstrated that immediately following the fusion of sperm and oocyte, soluble activating factors are released from the sperm into the oocyte cytoplasm [17,41,42,43]. Supporting evidence for the SF theory comes from studies showing that injection of soluble extracts from sperm into mammalian oocytes induces Ca^2+^ oscillations similar to those observed during fertilization [42,44]. The sperm factor appears to be a protein [42], as evidenced by studies showing that injection of soluble sperm extract activates mouse oocytes, leading to their development at least to the blastocyst stage [43]. Importantly, this sperm factor is not species-specific among mammals [44]. Further supporting this hypothesis, the injection of soluble sperm extracts into oocytes has been shown to elicit a rise in Ca^2+^ levels similar to that observed during fertilization across various species, including sea urchins [45,46]. Thus, it is proposed that one or more SFs exist in the sperm, and these SF(s) may serve as the trigger for inducing the increase in Ca^2+^ levels and subsequent oocyte activation. While various candidate factors have been reported, subsequent studies have shown that oscillin and tr-kit, initially identified as SFs, do not fulfill this role [47,48]. However, it was later revealed that sperm extracts contain a sperm-specific phospholipase C (PLC), which exhibits distinctive properties such as higher Ca^2+^ sensitivity compared to known PLC isoforms [49,50].

A novel sperm-specific PLC named phospholipase C zeta (PLCζ) (gene name: *Plcz1*) was eventually identified [51], and it is considered to have all of the requirements of an SF for oocyte activation. PLCζ has been detected in mice [51,52], rats [53], humans [53,54], macaque monkeys [54], cows [55], pigs [56,57], horse [58,59], quail [60], and medaka (Japanese rice fish) [53]. *Plcz1* is a gene that is widely conserved among animal species, and it is now known that PLCζ hydrolyzes phosphatidylinositol 4,5-bisphosphate (PIP_2_) to IP_3_ [51,61], which binds to the receptor of IP_3_ (i.e., IP_3_R) on the endoplasmic reticulum (ER) and releases Ca^2+^ stored in the ER into the oocyte cytoplasm, causing an increase in Ca^2+^ in the oocyte at fertilization [31,62,63].

Microinjection of a small amount of *Plcz1* mRNA (0.02 mg/mL) into mouse oocytes induced Ca^2+^ oscillations similar to those observed during IVF, and 62% of the embryos reached the morulae/blastocyst state [51]. The amount of PLCζ that can induce Ca^2+^ oscillations (4–75 fg, i.e., 0.002–0.02 mg/mL cRNA) is in a range that is similar to that of the PLCζ protein in a single sperm (20–50 fg) [51]. On the other hand, when transgenic mice suppressing the expression of PLCζ mRNA were generated, PLCζ protein was reduced in their sperm, resulting in reduced Ca^2+^ oscillations activity and oocyte activation [64]. These findings suggest that PLCζ plays important roles in oocyte activation and embryonic development during mammalian fertilization.

**Figure 4:**
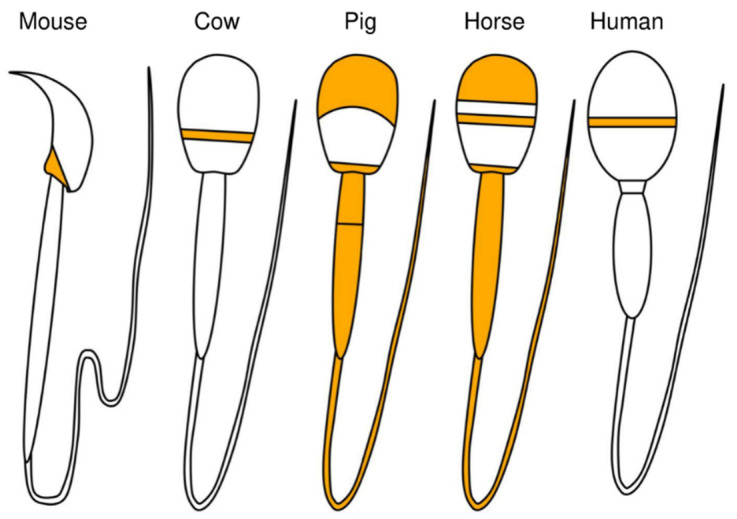
Species-dependent localization of PLCζ in mammalian spermatozoa. Orange indicates the localization of PLCζ protein.

Regarding the localization of PLCζ in mice, PLCζ was shown to be located in the post-acrosomal region of the sperm [65] (Figure 4). In bovine sperm, PLCζ was observed in the equatorial region [65]; in porcine sperm, PLCζ was observed in both acrosomal and post-acrosomal regions and the tail [56,66]; and in horse sperm, PLCζ was observed in the apical body, equatorial region, head–midpiece junction, and tail, indicating differences among animal species [58,59]. It was reported that changes in the localization of PLCζ in sperm are influenced by phenomena such as AR. AR is an exocytotic process that is essential for mammalian fertilization and is critical for the release of acrosomal contents into the oviduct and for the process that allows sperm to interact and fuse with the oocyte [67]. In humans, after fertilization or AR, the proportion of sperm with PLCζ protein localized to the equatorial/acrosomal region decreased, and the proportion with PLCζ localized to the posterior equatorial/acrosomal region increased [68]. It is not clear whether PLCζ protein actually moves to the posterior acrosomal region during fertilization and/or AR. Taken together, the above-described findings indicate that the localization of PLCζ in sperm, which varies among animal species and fertilization events, may be involved in species-specific fertilization mechanisms.

## 2. The Functions of PLCζ

### 2.1. The Molecular Structure of PLCs

**Figure 5:**
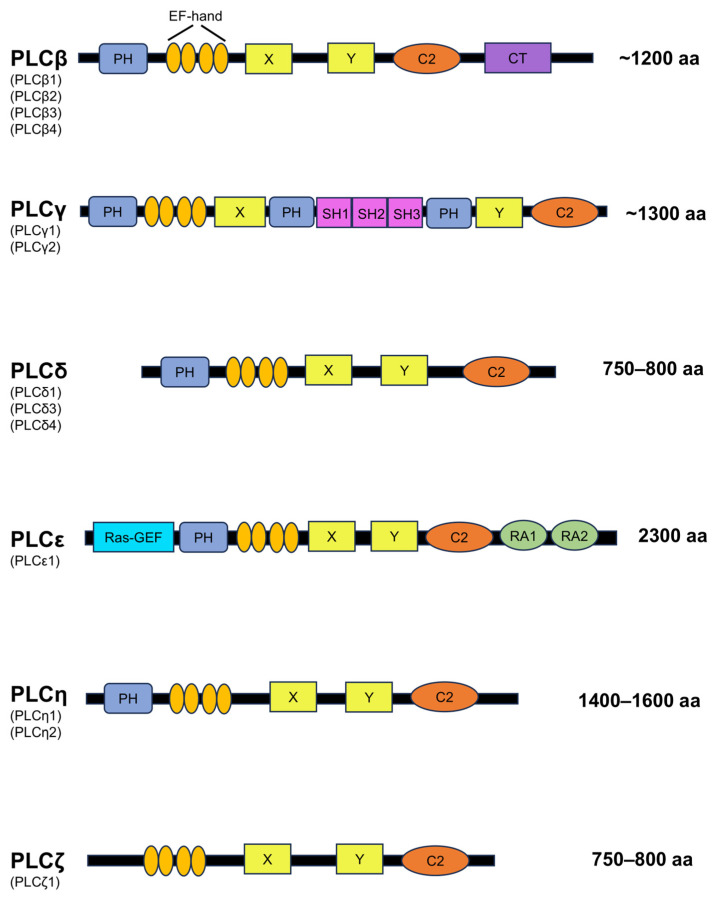
Schematic diagram of PLC domains in mammals. Different color indicates different domain.

Six isotypes (PLCβ, PLCγ, PLCδ, PLCε, PLCζ, and PLCη) and 13 subtypes (β1-4, γ1-2, δ1, δ3-4, ε1, ζ1, and η1-2) of PLCs are currently known in mammals (Figure 5) [69,70]. The structures of these PLCs have in common the pleckstrin-homology (PH domain) (except for PLCζ), an EF-hand domain, an X-Y catalytic domain, and a C2 domain [30,51]. The PH domain is composed of seven β-sheets and an α-helix, and the β-sheet of the PH domain binds to PIP_2_ [71,72]. The β-sheet of the PH domain has been shown to bind to PIP_2_ [71]. The PH domain is a structure that plays an important role in the binding of PLC to PIP_2_ and G proteins at the plasma membrane [73]. The EF-hand motif contains a helix–loop–helix structure, which is also present in numerous Ca^2+^-binding proteins such as myosin, calmodulin, calreticulin, and troponin [74]. After binding with Ca^2+^, the EF-hand motif alters its conformation and triggers Ca^2+^-regulatory functions by disclosing the ligand sites on other proteins. This process stabilizes the PLC structure [75]. Irrespective of the concentration of Ca^2+^, removal of the EF-hand motif from the enzyme diminishes the function of PLC [76]. When Ca^2+^ binds to the EF-hand motif, it triggers the binding of PLC to PIP_2_ through the PH domain [72,76]. The catalytic X-Y domain comprises approximately 300 amino acids and is situated on the C-terminus of the EF-hand motif [72]. These domains, also known as triosephosphate isomerase (TIM) barrel domains, are composed of alternating α-helices and β-sheets, forming a βaβaβaβ pattern with a TIM barrel-like structure [72]. The X region, which harbors all catalytic residues, is moderately conserved among members of the PLC family [72,77]. The X and Y catalytic domains, also known as the TIM barrel-like domain, are responsible for the catalytic activity of PLC [69,78]. According to a study using two cyclic inositol 4,5-bisphosphate, the catalytic activity of this domain increases as the concentration of Ca^2+^ increases from 0.01 μM to 10 μM [78]. The Y domain, which comprises residues 489–606, forms the other half of the TIM barrel-like structure [79]. The eightfold barrel structure is found in enzymes that regulate metabolism, with diverse functions [79]. The Y domain plays a critical role in substrate recognition, regulating PLCs’ preference for PIP_2_, PIP, and PI [69]. The X-Y domains of PLC isoforms are separated by an X-Y linker sequence [54], which is believed to be essential. The X-Y linker sequence of PLC is negatively charged, maintaining the inactive state of the PLC [51]. Upon stimulation, PLC translocates to the plasma membrane [80]. The negatively charged plasma membrane and X-Y linker sequence repel each other, leading to structural changes in PLC and enabling its binding to PIP_2_ [80].

The C2 domain, found at the C-terminus, consists of approximately 120 amino acids [81] and is present in more than 40 proteins, including protein kinase C [72]. The eight-stranded antiparallel beta sandwich forms the C2 domain in PLC family members [72]. The presence of multiple binding sites for phospholipids, dependent on Ca^2+^ in the C2 domain, suggests that the binding sites work synergistically [82]. The EF-hand motif possesses a Ca^2+^-binding pocket formed by successive stretches of amino acids in the primary sequence [83,84]. Conversely, the Ca^2+^-binding pockets of the C2 domain reside far apart from each other in the amino acid sequence associated with the C2 domain and are separate from each other [83,84]. Functionally, the EF-hand motif, considered the most prevalent Ca^2+^-binding motif in proteins, could potentially compete with the C2 domain for Ca^2+^ binding [85].

### 2.2. Features of PLCζ

PLCζ is the smallest PLC of all PLCs studied to date and maintains high hydrolytic ability (Figure 5). Its unique structure and properties distinguish it from other members of the PLC family, which we will discuss later in this chapter. One notable feature of PLCζ is its absence of a PH domain, distinguishing it from all other members of the PLC family [51]. Although the PH domain enables PLCs to link with components of cell membranes, including PIP_2_ and G proteins [71], PLCζ moves into the cytoplasm and binds with PIP_2_ situated within tiny cellular sacs, thereby enhancing its remarkable ability to break down PIP_2_ molecules after the fusion of sperm and oocyte [86]. PLCs, except PLCζ, perform membrane targeting of PIP_2_ through the PH domain; PLCζ lacks the PH domain, and membrane targeting of PIP_2_ is performed by the EF-hand domain. This was demonstrated by the fact that the introduction of an EF-hand domain mutant PLCζ protein into mouse oocytes reduced PIP2 hydrolysis without affecting Ca^2+^ sensitivity [87].

In addition, compared to other PLCs, PLCζ has high activity even at very low Ca^2+^ concentrations [30]. Ca^2+^ sensitivity is largely attributed to the EF-hand domain, which comprises four motifs located at the N-terminus of PLCζ and is primarily responsible for Ca^2+^ regulation [88,89]. The deletion of EF1, EF2, and EF1-2 had no effect on Ca^2+^ sensitivity, but the EF1-3 deletion in PLCζ mutants required 12 times more Ca^2+^ than PLCζ to degrade PIP_2_ as much as PLCζ degrades PIP_2_; in addition, a marked decrease in Ca^2+^ sensitivity was observed [90]. Thus, the characteristic Ca^2+^ sensitivity of PLCζ is likely to involve the structural determinant EF1-3. However, even the Ca^2+^ sensitivity of ΔEF1-3 PLCζ mutants was observed to be 15-fold higher than that of PLCδ1, suggesting that EF4 of PLCζ may also be involved in the high Ca^2+^ sensitivity of PLCζ [90]. EF1 and EF2 of PLCζ contain Ca^2+^-binding loop sequences that are homologous to the Ca^2+^-binding site [76]. PLCζ mutants in which Asp (an amino acid residue that is present in the Ca^2+^-binding loop sequence) was mutated to Arg did not show reduced Ca^2+^ sensitivity [90]. This result is consistent with the above-described finding that the ΔEF1, ΔEF2, and ΔEF1-2 PLCζ mutants do not reduce Ca^2+^ sensitivity, indicating that the EF1 and EF2 Ca^2+^-binding loop sequences of PLCζ are not involved in the Ca^2+^ sensitivity of PLCζ.

An injection of PLCζ mutant cRNA lacking one or both pairs of the EF-hand domains of PLCζ into mouse oocytes under the same conditions as those used for wildtype (WT) PLCζ significantly reduced Ca^2+^ oscillations [89]. In the evaluation of the rate of pronuclear formation when ΔEF1-3 PLCζ cRNA was injected into the oocyte, WT PLCζ showed a high rate of pronucleus formation at 0.002 mg/mL, but ΔEF1-3 PLCζ could hardly induce pronuclear formation. However, when 1 mg/mL of ΔEF1-3 PLCζ cRNA was injected, the rate of PN formation was similar to that of WT PLCζ [91]. These results indicate that a reduction in the EF-hand motif reduces Ca^2+^ sensitivity, but not completely. Thus, the EF-hand domain of PLCζ is responsible for membrane targeting of PIP_2_ instead of the PH domain. Although a single EF-hand motif does not cause the loss of Ca^2+^ sensitivity, the characteristic high Ca^2+^ sensitivity of PLCζ is regulated by the EF-hand domain, which consists of four EF-hand motifs.

The TIM barrel domain of PLCζ, consisting of the X and Y catalytic domains, is located between the EF-hand domain and the C2 domain and is the part that is actually responsible for enzyme activity [51]. Several cases of X-Y catalytic domain mutations have been reported in human male infertility; the X and Y catalytic domains of PLCζ are similar to sequences of other PLCs, and most of the sequences are conserved [51]. Saunders et al. [51] reported that replacing Aspartic Acid at position 210 (D210) (which is involved in the catalytic reaction at the active site residue of PLCδ1 and is conserved in the X catalytic domain of PLCζ [92,93]) with arginine (D210R) resulted in the loss of Ca^2+^ vibration-inducing activity. Aspartic Acid at position 210 corresponds to Aspartic Acid at position 343 in PLCδ1, and like PLCδ1, this amino acid residue is involved in the Ca^2+^-binding site that is responsible for enzyme activity in PLCζ [92,94]. In addition, the replacement of both the K299 and K301 of PLCζ with glutamic acid (K299A or K301A) resulted in a loss of Ca^2+^ vibration-inducing activity [95]. These amino acid residues correspond to K432 and K434 in PLCδ1 and are similar to the sites that interact with the substrate PIP_2_ [96,97], and it is possible that these mutations render interaction with PIP_2_ impossible. PLCζ is known to move around the nucleus when PN formation occurs after the induction of Ca^2+^ oscillations [98], but the base substitutions in D210R, K299A, and K301A inhibited the nuclear migration of PLCζ after PN formation. This indicates that the X catalytic domain of PLCζ has a role not only in enzymatic activity but also in nuclear translocation.

PLCζ has a long X-Y linker, i.e., an unstructured region mediated by the X and Y catalytic domains, which is one of the characteristic structures of PLCζ [69] and has been shown to play several roles [80,95,99]. The X-Y linker regions of PLCβ, PLCγ, PLCδ, and PLCη are negatively charged, whereas the linker of PLCζ is positively charged [80]. The positively charged X-Y linker sequence of PLCζ facilitates binding to the negatively charged PIP_2_ and is one of the factors responsible for the very high hydrolytic resolution of PLCζ [80]. In mouse experiments, the deletion of the X-Y linker of PLCζ markedly reduced the ability of PLCζ to induce Ca^2+^ oscillations [95]. In addition, when three of the lysines in the X-Y linker sequence were replaced with alanine (K374A, K375A, and K377A) in mice, the positive charge of the linker portion decreased, and these mutations have been shown to reduce the effectiveness of the PIP_2_ interaction and Ca^2+^ oscillations-inducing activity in vitro [99]. The X-Y linker also contains a nuclear localization sequence (NLS) for nuclear migration, which is thought to prevent long-lasting and excessive Ca^2+^ oscillations after pronuclear formation by inducing PLCζ into the nucleus [95]. The X-Y linker, together with the EF-hand domain, is thus considered essential for efficiently promoting PIP_2_ binding and maintaining the high hydrolytic resolution of PLC [80,99]. However, various functions are thought to be retained in different mammalian species, such as porcine PLCζ, which retains its biological activity even after proteolytic cleavage of the X-Y linker [100]. To elucidate the essential role of the X-Y linker of PLCζ in mammals, further studies are required. 

The C2 domain of PLCζ is located at the C-terminus and is involved in the targeting of proteins to the plasma membrane. PLCs except PLCζ bind to PIP_2_ on the plasma membrane, but PLCζ showed minimal loss of PIP_2_ on the oocyte membrane even after Ca^2+^ oscillations, and depletion of the oocyte membrane PIP_2_ pool had no effect on Ca^2+^ oscillations. The C2 domain of PLCζ is largely responsible for this. PLCs other than PLCζ are targeted to PIP_2_ by the PH domain [78]. The C2 domain binds to phospholipid-containing membranes and exhibits different phospholipid selectivity [101], suggesting that PLCζ may target and specifically hydrolyze PIP_2_ in the plasma membrane by its C2 domain instead of its PH domain [99]. In vitro experiments have also shown that the C2 domain of PLCζ interacts with PI(3)P and PI(5)P in a Ca^2+^-independent manner and inhibits their binding to PI(4,5)P_2_ [90]. This interaction with PI(3)P and PI(5)P may play an important role in the inhibition of enzyme activity by PLCζ before fertilization [90]. Deletion of the C2 domain from PLCζ results in a loss of Ca^2+^ oscillations-inducing activity even though the Ca^2+^ sensitivity is not affected [102]. In addition, replacing the C2 domain of PLCζ with the C2 domain of PLCδ1 did not cause a release of Ca^2+^ [102]. This experiment also suggested that the C2 domain of PLCζ binds to PIP_2_ for hydrolysis. However, in the membrane-spotted arrays performed by Nomikos et al. [99], the binding of PIP_2_ to the C2 domain of PLCζ was very weak, while PIP_2_ showed strong binding to the X-Y linker. It is thus suggested that the binding of PLCζ to PIP_2_ is carried out mainly by the X-Y linker, although the C2 domain is also responsible.

Deletion of the C2 domain of PLCζ results in a loss of Ca^2+^ oscillations induction activity [102], which plays an important role in the induction of Ca^2+^ oscillations by PLCζ but may retain an important unknown function other than PIP_2_ binding. It was reported that PLCζ mutant proteins lacking the C2 domain of PLCζ or the 37 amino acid residues in the C-terminal region adjacent to the C2 domain (PLCζ 611–647) were completely unable to hydrolyze PIP_2_ at all in the presence of Ca^2+^ and completely lose PLC activity [90]. The C-terminal region (as well as the C2 domain) is essential for the induction of Ca^2+^ oscillations in PLC activity; the C2 domain of PLCζ is known to bind to the N-terminal EF-hand domain [95]. The binding of the C2 domain to the EF-hand domain exposes the X-Y domain of PLCζ, making the catalytic activity of PIP_2_ more efficient [95]. For these reasons, the C2 domain plays an important role in the regulation of the enzymatic activity of PLCζ at the appropriate time and in the high enzymatic activity due to the conservation of the C2 terminus.

### 2.3. Mutations and Manipulations of PLCζ

Several studies have used transgenic (Tg) and knockout mice to clarify the functions of PLCζ in mammalian fertilization. In 2005, researchers generated *Plcz1*-RNAi Tg mice that express *Plcz1* short hairpin (sh)RNA to inhibit PLCζ function [64]. The sperm derived from the *Plcz1*-RNAi male mice exhibited neither morphological nor motility abnormalities, but the number of Ca^2+^ oscillations was greatly decreased when the sperm were used for IVF [64]. When in vivo fertilized oocytes were collected from the oviduct and cultured after mating *Plcz1*-RNAi male mice with WT female mice, the fusion of sperm and oocyte and the extrusion of the second polar body were similar to those of the controls, but oocyte activation did not occur, and the blastocyst development rate was reduced (control: 99% vs. *Plcz1*-RNAi Tg: 65%) [64]. The fertility of the *Plcz1*-RNAi male mice was also decreased (7.0 ± 0.6 pups/litter) compared to the controls (12.0 ± 1.3 pups/litter) [64]. In addition, offspring derived from *Plcz1*-RNAi male mice were not obtained [64]. These findings indicate that PLCζ plays a critical role in inducing Ca^2+^ oscillations and further embryonic development, at least in mice.

In 2007, *Plcz1*-Tg mice with a CAG promoter were generated for PLCζ overexpression [103]. The litter size was dramatically decreased in some strains derived from *Plcz1*-Tg male mice after mating with female mice (WT: 7.91 ± 0.29, Tg: 0.85 ± 0.348) [103]. When germinal vesicle (GV)-stage oocytes were collected from the ovaries of *Plcz1*-Tg female mice, the oocytes underwent spontaneous germinal vesicle breakdown (GVBD) and developed to the MII stage [103]. During the progression to the MII stage, increases in Ca^2+^ were observed in the oocytes [103]. The oocytes derived from PLCζ mice after mating with WT female mice showed segregation of metaphase chromosomes and the completion of meiosis, resulting in the induction of parthenogenetic activation and development to the blastocyst stage [103]. On the other hand, the ovaries of female mice mated with *Plcz1*-Tg males exhibited tumorigenesis [103]. From the results described above, it is apparent that the optimal timing and the optimal amount of PLCζ activity are essential for oocyte activation and further embryonic development.

Although the importance of PLCζ was demonstrated in the two aforementioned studies, it remains unclear whether PLCζ is indispensable for mammalian fertilization. Nearly two decades after the discovery of PLCζ in 2002, the generation of PLCζ gene-deficient (*Plcz1^−/−^*) mice has been reported by two different research groups [32,33]. Histological analysis of testes from *Plcz1^−/−^* male mice demonstrated that the loss of *Plcz1^−/−^* had no adverse effect on spermatogenesis [32]. Epididymal sperm from *Plcz1^−/−^* male mice also showed normal viability, motility, and acrosome reaction [32]. In summary, it has been shown that a loss of *Plcz1* had no apparent effect on spermatogenesis or the ability of sperm to bind and fuse with oocytes.

They also investigated whether the application of ICSI with *Plcz1^−/−^* sperm could induce Ca^2+^ oscillations in the oocyte; their findings revealed that Ca^2+^ oscillations in the oocyte did not occur at all in the embryos that were fertilized with *Plcz1^−/−^* sperm [32,33]. These results suggest that PLCζ regulates Ca^2+^ oscillations in the oocyte during fertilization. The researchers also performed IVF with *Plcz1^−/−^* sperm. Unexpectedly, oocytes fertilized with *Plcz1^−/−^* sperm showed fertility that was similar to that of WT oocytes [32,33]. *Plcz1^−/−^* sperm-derived fertilized oocytes also exhibited a high rate of polyspermy [32,33]. In addition, fertilized oocytes from the oviducts of WT female mice mated with male *Plcz1^−/−^* mice were obtained, and the rate of polyspermy was assessed. The results revealed that, similar to IVF, the fertilized oocytes derived from *Plcz1^−/−^* male mice had an increased rate of polyspermy [32,33]. An increase in the number of pronuclei was observed in some oocytes fertilized by *Plcz1^−/−^* sperm, suggesting delayed fertilization and oocyte activation by *Plcz1^−/−^* sperm. The blastocyst development rate of oocytes fertilized by *Plcz1^−/−^* sperm was significantly decreased (WT 90.2%, *Plcz1^−/−^* 32.2%) [33]. These results suggest that the suppression of polyspermy is not working properly due to *Plcz1* deficiency. It could have been proposed that (i) Juno, expressed on the oocyte surface, is indispensable for preventing polyspermy [104], and (ii) PLCζ may have a role in the mechanism underlying the blocking of polyspermy. Those authors also observed an improvement in the blastocyst development rate of fertilized oocytes derived from *Plcz1^−/−^* sperm by injection of *Plcz1* cRNA or mRNA after ICSI [32,33]. This indicates that *Plcz1* also plays an important role in embryonic development.

The number of pups per litter produced by the *Plcz1^−/−^* male mice was significantly lower than that of the WT, but the *Plcz1^−/−^* male mice were still fertile (WT 7.8 ± 0.8 vs. *Plcz1^−/−^* 3.2 ± 1.2~4.2 ± 0.6 pups/litter [32]; WT 8.9 ± 0.26 vs. *Plcz1^−/−^* 2.3 ± 0.50 pups/litter [33]). It was thus demonstrated that embryos derived from *Plcz1^−/−^* male mice were able to develop to term without Ca^2+^ oscillations by PLCζ, although defects such as oocyte activation failure (OAF) and polyspermy reduced the number of pups per litter. Nozawa et al. [33] also performed IVF with the use of various sperm concentrations (2, 10, and 50 × 10^3^ sperm/mL) to further investigate the fertilization and oocyte activation potential of *Plcz1^−/−^* sperm. In *Plcz1^−/−^* sperm, activation failure was more pronounced at the lowest sperm concentration (WT 0% and *Plcz1^−/−^* 12.4% at 2 × 10^5^/mL), and the polyspermy rate increased to about 80% at the highest sperm concentration (WT 7.6% and *Plcz1^−/−^* 82.4% at 50 × 10^3^/mL) [33]. When the intracellular Ca^2+^ concentration was monitored during IVF using live imaging, all single-sperm fertilized eggs with *Plcz1^−/−^* sperm showed rise(s) in intracellular Ca^2+^ regardless of PN formation. However, these fertilized oocytes had a significantly reduced number of Ca^2+^ spikes (WT 12.0 ± 5.68 spikes vs. *Plcz1^−/−^* 2.75 ± 0.65 spikes) [33]. As the number of sperm fusing increased, the number of Ca^2+^ spikes increased, and more fertilized oocytes resumed the cell cycle. These results suggest that sperm have PLCζ-independent oocyte activation ability. However, a single sperm is insufficient for oocyte activation to cause resumption from the MII arrest, and incomplete oocyte activation may lead to polyspermy.

A previous study introduced two mutations into mice, i.e., H435P (a mutation corresponding to human H398P) and D210R (an enzymatic dead mutation), in order to investigate the mechanism of infertility caused by point mutations in *Plcz1* reported in humans [33]. An immunoblot analysis of sperm proteins revealed that the WT and D210R PLCζ mutation exhibited an approximately 74 kDa signal, whereas an approx. 20 kDa signal was detected only in the sperm of the H435P PLCζ mice used in the experiment [33], suggesting instability of the H435P protein in vivo. These results indicated that H435P PLCζ seems to be unstable in vivo. The spermatogenesis, fertility, IVF, and ICSI results obtained with homozygous male mice with the D210R or H435P PLCζ mutation were comparable to those of *Plcz1^−/−^* male mice [33]. ICSI with these mouse sperm and the microinjection of mouse and human *Plcz1* mRNA resulted in Ca^2+^ oscillations in the oocyte and 2PN formation [33]. These embryos transplanted into pseudopregnant mice yielded normal litters.

As described above, the importance of PLCζ in oocyte activation was demonstrated using *Plcz1^−/−^* mice by two research groups, and both groups also demonstrated that *Plcz1^−/−^* sperm caused polyspermy due to failure of the zona reaction [32,33], suggesting a new role for PLCζ. Although polyspermy has been reported in male infertility after IVF, the cause of this phenomenon has been unknown; the present results indicate a possible abnormality of PLCζ in the sperm of these individuals.

### 2.4. Human PLCζ

Currently, in infertility treatment, if there is no pronucleation in multiple cycles of ICSI or ROSI, it is considered to be a failure of oocyte activation [105]. In humans, male infertility has been reported due to PLC-zeta abnormalities. The abnormalities of PLCζ are thought to be one of the causes of the failure of oocyte activation [106].

Therefore, PLCζ has recently gained attention as an infertility diagnostic tool [107]. Artificial oocyte activation was the only treatment for oocyte activation failure. As mentioned above, artificial oocyte activation has been used when multiple cycles of in vitro fertilization or ICSI have failed to achieve fertilization or when low fertilization rates have been observed, and there are no tools to diagnose sperm–oocyte activation failure. By performing a fluorescence analysis of PLCζ and observing its fluorescence level and localization pattern, it is possible to predict the gene expression level and localization of PLCζ and indirectly diagnose whether artificial oocyte activation can improve sperm fertilization rates in male infertility patients. This measurement can avoid unnecessary IVF and reduce the consumption of unwanted oocytes.

Infertility caused by mutations in the PLCζ gene has also been reported in humans, and various phenotypic abnormalities have been reported with mutations. The mutation (H233L or H398P) of PLCζ found in infertile male humans altered the localization of PLCζ, although these mutations did not affect sperm morphology [104,108]. The microinjection of these mutant *Plcz1* cRNAs into oocytes also resulted in a marked reduction in the number and amplitude of Ca^2+^ oscillations [101]. PLCζ (H233) is located in exon 6 and encodes the X catalytic domain, while H389 is located in exon 11 and encodes the Y catalytic domain [109]. The X-Y catalytic domain is the domain responsible for enzyme activity. An injection of patient sperm into mouse eggs reduced Ca^2+^ oscillations. This was the first finding that base substitutions in the X-Y catalytic domain in human sperm altered the localization of PLCζ in the spermatozoa [109]. They then expressed H233L and H398P PLCζ with the use of human embryonic kidney (HEK)293T cells, injected the cells into oocytes, and measured their cytoplasmic localization. They then observed that the expression levels were decreased, suggesting that genetic mutations due to base substitutions were responsible for the instability of the proteins [110].

The sperm of two infertile brothers reported by Escoffer et al. in 2016 both carried the missense mutation I489P PLCζ, and their sperm showed abnormal localization in the oocyte after ICSI and a significant decrease in Ca^2+^ oscillations in the oocytes [111]. PLCζ I489 is located in exon 13 and encodes the C2 domain. In addition, embryos fertilized via ICSI using the brothers’ sperm failed to develop to the two-cell stage. These events suggest that PLCζ in human spermatozoa also acts in the same way to induce Ca^2+^ oscillations by the hydrolysis of PIP_2_ localized in the plasma membrane, a feature of the specific phospholipid selection of the C2 domain performed using mice [111].

All previous *Plcz1* mutations have been single missense mutations, but compound heterozygous mutations in *Plcz1* were identified in an infertile male patient [112]. This patient was found to have a P420L substitution due to a base substitution in 1259C>T and an M578T residue substitution due to a base substitution in 1733T>C. Sequencing of the parental DNA of the patient revealed that the father was a carrier of 1733T>C (M578T), and the mother was a carrier of *Plcz1* 1259C>T (P420L).

Artificial oocyte activation procedures such as Ca^2+^ ionophores are performed when oocyte activation failure is observed [113]. Mutations of *Plcz1* in human sperm reduce oocyte activation, indicating male infertility, and fertility was restored in most of these individuals after ICSI by an injection of *Plcz1* RNA or an activation treatment such as Ca^2+^ ionophore and SrCl_2_ [113,114]. However, in 2019, Torra-Massana et al. reported that out of 12 patients with genetic mutations in *Plcz1* (residue substitutions at R197H and H233L in the X catalytic domain and a deletion mutation of two bases of V326K in the X-Y linker), the sperm from three patients did not undergo mouse oocytes activation after receiving an injection of *Plcz1* cRNA. This finding suggests that mutant *Plcz1* may also impact the enzymatic activity of normal PLCζ [115].

**Figure 6:**
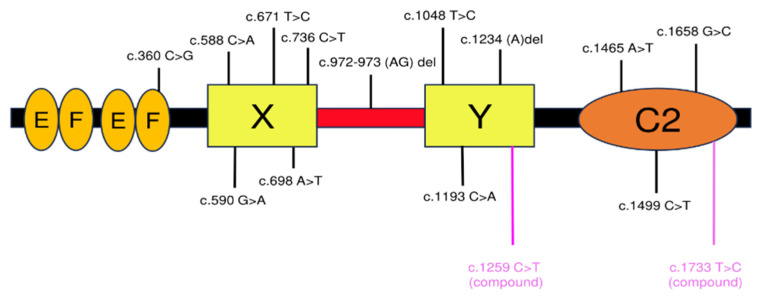
Summary of PLCζ mutations identified by the scientific literature. Schematic diagram of PLCζ domains with the mutation. The positions of the variants identified reported in the literature. Pink indicates compound heterozygous mutations.

Currently, 1–3% of infertile male patients are reported to have reduced oocyte activation ability [116], and sperm from these individuals fail to activate oocytes and produce pregnancy even with ICSI. Since there have been many reports of severely reduced oocyte activation ability due to *Plcz1* mutations [105,117], further research on *Plcz1* mutations in human sperm could improve fertility treatments for infertile couples with *Plcz1* gene mutations. Table 1 and Figure 6 described the major *Plcz1* mutations and their phenotypes reported in humans to date [118,119,120].

**Table 1:** Summary of human *Plcz1* variants.

Mutation (Protein)	Mutation (*PLCZ1* Coding Sequence)	Gene Location	Protein Location	Type of Mutation	Phenotype	Authors	Date
p.I120M	c.360 C>G	Exon 4	EF-hand	Missense	OAF	Torra-Massana et al.	2019 [115]
p.C196 *	c.588 C>A	Exon 6	X	Missense	OAF	Dai et al.	2020 [118]
p.R197H	c.590 G>A	Exon 6	X	Missense	OAF	Torra-Massana et al.	2019 [115]
p.L224P	c.671 T>C	Exon 6	X	Missense	OAF	Torra-Massana et al.	2019 [115]
p.H233L	c.698 A>T	Exon 6	X	Missense	OAF	Kashir et al.	2011 [110]
p.L246F	c.736 C>T	Exon 7	X	Missense	OAF	Dai et al.	2020 [118]
p.V326K fs	c.972-973 (AG) deletion	Exon 6	X-Y linker	Frameshift	OAF	Torra-Massana et al.	2019 [115]
p.S350P	c.1048 T>C	Exon 10	Y	Missense	OAF	Dai et al.	2020 [118]
p.H398P	c.1193 C>A	Exon 11	Y	Missense	OAF, protein instability	Heytens et al.	2009 [108]
p.R412fs	c.1234 (A) del	Exon 11	Y	Frameshift	OAF	Mu et al.	2020 [119]
p.I489P	c.1465 A>T	Exon 13	C2	Missense	OAF	Escoffier et al.	2016 [111]
p.S500L	c.1499 C>T	Exon13	C2	Missense	OAF	Torra-Massana et al.	2019 [115]
p.R553P	c.1658 G>C	Exon 14	C2	Missense	OAF, protein instability	Yuan et al.	2020 [120]
p.P420L	c.1259 C>T (compound)	Exon 12	Y	Missense	OAF	Yuan et al.	2020 [112]
p.M578T	c.1733 T>C (compound)	Exon 14	C2	Missense	OAF	Yuan et al.	2020 [112]

The asterisk indicates the stop codon.

## 3. Future Directions for a Better Understanding of Oocyte Activation

### 3.1. Another Candidate Factor(s) Inducing Oocyte Activation

Although PLCζ has been considered a potent SF, it remains uncertain whether it is the sole factor and whether it is essential for Ca^2+^ oscillations and oocyte activation. Several research groups have reported possible alternative sperm factor candidates. In 2007, Oko’s group discovered a protein in the post-acrosomal sheath region of the sperm head’s perinuclear theca (PT), and the protein was extractable under alkaline conditions [121]. This protein shares sequence homology with the N-terminal region of the WW domain-binding protein 2 (WBP2), and its C-terminal region is substantially enriched in proline [121]. Post-acrosomal sheath WW domain-binding protein (PAWP) is the designated name for the protein, which is also known as WBP2NL [121]. The impact of introducing recombinant human PAWP or alkaline PT extract into MII-arrested oocytes from porcine, cows, macaques, and Xenopus was examined [121], and the results revealed a high rate of PN formation in the oocytes after either injection, indicating that PAWP has potential as a SF for oocyte activation.

The ability of PAWP to induce Ca^2+^ increases during oocyte activation is a crucial factor in assessing its candidacy as a sperm factor. A study by Aarabi et al. (2009) [122] showed that Xenopus oocytes injected with recombinant human PAWP resulted in an intracellular release of Ca^2+^. Their subsequent research established that introducing human PAWP protein through an injection elicits Ca^2+^ oscillations and PN formation in mammalian oocytes (both mouse and human) [123]. These results strongly suggested that PAWP is a sperm factor, like PLCζ. Another SF candidate is extra-mitochondrial citrate synthase (eCS), which was first identified as a sperm factor in *Cynops pyrrhogaster* [124]. In the mouse, eCS was localized in the sperm head, and an injection of *eCs* mRNA induced Ca^2+^ oscillations in the oocyte [125], suggesting that eCS also appears to meet the requirements as an SF. Subsequent studies using gene-deficient mice have shown that PAWP and eCS are not essential for Ca^2+^ oscillations and oocyte activation [125,126].

However, the possibility that there may be factors involved in oocyte activation beyond PLCζ cannot be completely ruled out. Indeed, results obtained in studies of *Plcz1^−/−^* mice suggest that there may be other unknown mechanisms that induce the rise in intracellular Ca^2+^ because a few oscillations of intracellular Ca^2+^ were still observed in the oocytes when *Plcz1^−/−^* sperm were applied for IVF but not for ICSI, suggesting that the underlying mechanism requires the membrane fusion between sperm and oocyte [33]. As mentioned above, there is no doubt that the sperm factor theory is currently the main pathway for the rise(s) in Ca^2+^ during fertilization, but the other two theories may need to be reconsidered.

In addition, the results from the study using *Plcz1^−/−^* have led us to the possibility of the presence of not only “sperm factor” but “Oocyte factor” for membrane fusion-mediated Ca^2+^ oscillations. This is because Ca^2+^ oscillations require specific PIP_2_ conditions: as described above, PLCs except for PLCζ target and hydrolyze PIP_2_ on the plasma membrane, whereas PLCζ hydrolysis PIP_2_ in the oocyte cytoplasm [86]. Furthermore, PIP_2_ fused to PLCζ-formed clusters in the oocyte cytoplasm, and Ca^2+^ oscillations decreased as the distance between PIP_2_ in the oocyte cytoplasm increased [127]. It was also found that the PLCζ protein did not show Ca^2+^ oscillations when injected into hamster ovary cells [128]. These results suggest a possibility that the Ca^2+^ oscillations that occur during fertilization may require currently uncharacterized factor(s) on the oocyte side and that “other factor(s)” is present besides PLCζ on the sperm side.

Based on the RNA sequence in germ cells, it may be informative to explore the possibility of PLCs other than PLCζ that are highly expressed in sperm and oocytes and that could be candidate factors inducing the rise in Ca^2+^. In mouse oocytes, PLCε1 is highly expressed compared to other PLCs, and therefore, we used mice lacking the *Plce1* gene (*Plce1^−/−^*), which were previously produced by another group [129,130]. In our preliminary study, we observed that the female *Plce1^−/−^* mice did not show any impaired fertility. In addition, the oocytes retrieved from female *Plce1^−/−^* mice were successfully fertilized after IVF, and these embryos developed to the blastocyst stage, as did those from control mice. Although we did not measure the levels of intracellular Ca^2+^ in *Plce1^−/−^* oocytes during fertilization, even the *Plcz1^−/−^* sperm and *Plce1^−/−^* oocytes were also successfully fertilized. These findings suggest that (i) PLCε1 in the oocytes is not involved in oocyte activation, and (ii) PLCε1 can be ruled out as the candidate factor.

**Figure 7:**
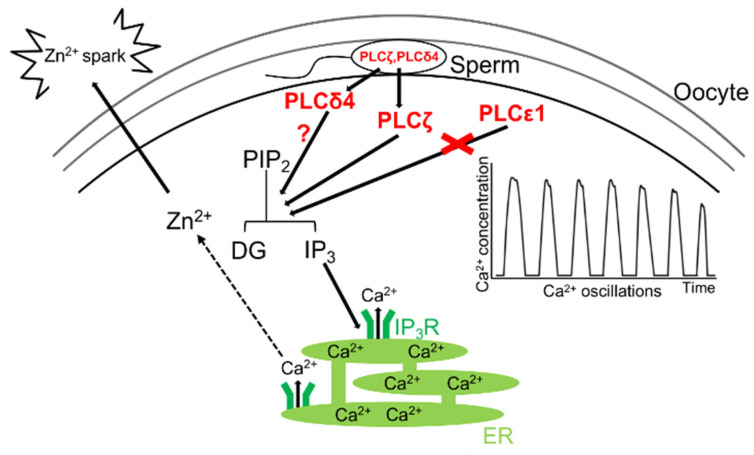
Proposed hypothesis of the mechanism underlying Ca^2+^ oscillations during mammalian fertilization.

PLCδ4 is one of the most abundantly expressed PLCs in spermatocytes [131], and PLCδ4-deficient (*Plcd4^−/−^*) male mice produced a low number of small litters or were sterile [131]. With the use of IVF, they also showed that insemination with *Plcd4^−/−^* sperm resulted in significantly fewer oocytes becoming activated and that the Ca^2+^ transients associated with fertilization were absent or delayed [131]. There is no doubt that PLCζ induces Ca^2+^ oscillations during mammalian fertilization [32,33], but nonetheless, PLCζ and PLCδ4 may cooperate to increase the fusion of membrane-dependent rises in intracellular Ca^2+^ and then initiate Ca^2+^ oscillations in oocytes during fertilization. Results from PLCζ and PLCδ4 double gene-deficient (*Plcz1^−/−^Plcd4^−/−^*) mice may provide answers to the questions described above (Figure 7).

### 3.2. Another Divalent Ion for Oocyte Activation

The fertilization process is widely acknowledged to be dependent on the increase in intracellular Ca^2+^ ions. However, recent investigations have illuminated alternative pathways for inducing meiotic resumption from the MII arrest. Notably, these pathways include the utilization of a Zn^2+^ chelator, TPEN, and the suppression of meiotic resumption through Zn^2+^ ionophore overload [132,133,134]. In mouse models, TPEN treatment alone was observed to effectively activate MII-arrested oocytes that had been injected with “inactivated” sperm heads, resulting in successful live births post-embryo transfer [133]. Intriguingly, this activation occurred in the absence of intracellular Ca^2+^ oscillations, challenging the established notion that full-term development is reliant on the release of Ca^2+^ during MII exit, as suggested by Suzuki et al. (2010) [133]. These findings collectively imply that while the elevation of intracellular Ca^2+^ ions in the oocyte is traditionally considered crucial for oocyte activation, the depletion of Zn^2+^ ions within the oocyte can also act as a trigger for activation.

When mature mouse oocytes, which are abundant in Zn^2+^, undergo fertilization, there is a transient release of Zn^2+^ into the extracellular milieu. This coordinated series of events has been termed the “Zn^2+^ spark” [135,136]. The occurrence of the Zn^2+^ spark was reported to coincide closely with the initial elevation of Ca^2+^ levels. Following its discovery in mice, the phenomenon of the Zn^2+^ spark has been observed in human and bovine oocytes subsequent to fertilization and oocyte activation in mice [135], humans [137,138], and cattle [139], suggesting a high degree of conservation across several mammalian species. Recently, the presence of the Zn^2+^ spark during fertilization was also confirmed in amphibian oocytes [140]. Despite the widespread occurrence of Zn^2+^ sparks, their exact role in fertilization remains incompletely understood. Bernhardt et al. proposed a comprehensive model suggesting that Zn^2+^ plays a crucial role in modulating the concentration-dependent regulation of meiosis through its interaction with Emi2, a Zn^2+^-binding protein and a key component of the cytostatic factor (CSF). According to their model, Zn^2+^ sparks facilitate the rapid and efficient inactivation of Emi2 [132]. It is noteworthy that the inactivation of Emi2 is known to occur through a Ca^2+^-dependent mechanism [141], and Zn^2+^ sparks are observed to be absent in the absence of Ca^2+^ chelation [137]. Furthermore, subsequent studies have provided additional insights into the effects of elevated Zn^2+^ levels on sperm function. One such study demonstrated that increased Zn^2+^ concentrations hinder the forward motility of sperm, thereby impeding their ability to traverse through the zona matrix and ultimately leading to the prevention of polyspermy [142]. These findings underscore the intricate interplay between Zn^2+^ signaling, Ca^2+^ dynamics, and sperm function during fertilization, highlighting the need for further investigation into the molecular mechanisms underlying these processes.

While further investigations are essential to comprehensively elucidate the intricacies of this mechanism, the significance of the Zn^2+^ spark during fertilization is widely acknowledged within the scientific community. Prior to the occurrence of a Zn^2+^ spark, there must be a substantial buildup of Zn^2+^ ions within the oocyte. Studies have consistently demonstrated that immature mouse oocytes exhibit an incapacity to generate a Zn^2+^ spark, thereby emphasizing the pivotal role of acute Zn^2+^ accumulation during meiotic maturation [137,143]. Moreover, the presence of Zn^2+^ accumulation has been well documented in MII oocytes [144]. The dynamic regulation of Zn^2+^ influx and efflux is governed by various Zn^2+^ transporters, such as ZIP1-14 and ZnT1-9. Notably, in mouse oocytes, the expression of ZIP6 and ZIP10 has been highlighted as significant [145]. Hence, it is hypothesized that Zn^2+^ influx in oocytes is intricately modulated by these ZIP proteins. By providing this detailed explanation, we aim to offer a more comprehensive understanding of the intricate processes surrounding the phenomenon of the Zn^2+^ spark during fertilization, shedding light on its role in reproductive biology and potential implications for assisted reproductive technologies.

Zn^2+^ spark profiles suggest that zygotes progressing into blastocysts release higher levels of Zn^2+^ compared to those that fail to develop further. This phenomenon correlates with increased rates of embryo development and a greater total cell number at the blastocyst [146]. Therefore, the levels of Zn^2+^ ions detected at specific time points of Zn^2+^ sparks could potentially serve as an early biomarker for assessing the quality of embryos in mouse models. These findings indicate that both the elevation of intracellular Ca^2+^ ions and the release of Zn^2+^ ions from the oocyte are essential for oocyte activation during fertilization. Furthermore, it is suggested that one or more sperm factors must be capable of inducing both of these events.

### 3.3. Conclusions

As previously discussed, the fertilization process in mammals involves complicated mechanisms, not all of which have been fully elucidated. Our proposed hypothesis for oocyte activation is shown in Figure 7. While the importance of the elevation of intracellular Ca^2+^ during oocyte activation is undeniable, it is believed that there are two distinct mechanisms of Ca^2+^ elevation within the oocyte: one involving unknown Ca^2+^ elevation through the fusion of sperm and oocyte membranes and the other involving Ca^2+^ oscillations mediated by PLCζ. Furthermore, further research is needed to elucidate the role of Zn^2+^ ion-dependent mechanisms, such as Zn^2+^ spark, in oocyte activation. The clarification of these Ca^2+^ and Zn^2+^-dependent oocyte activation mechanisms is expected not only to contribute to knowledge in the field of reproductive biology but also to facilitate research on the treatment of human infertility and the efficient reproduction of livestock and endangered wild animals.
